# Practical Notes on Diagnosis and Treatment

**Published:** 1907-02-23

**Authors:** 


### Practical Notes on Diagnosis and Treatment.

**Syphilitic Periostitis.**

In some cases of syphilis not only does mercury fail to prevent periostitis, but it appears to make it worse.—*Mr. Hutchinson.*

**Acute Dysentery.**

In acute dysentery or other irritable condition of the lower bowel, two to three drams of bismuth subnitrate given in an enema are much more useful than the same medicine ordered by the mouth.

**Theobromin as a Diuretic.**

Theobromin (45 to 75 grains daily for three days) is often very successful in cases of cardiac dropsy. It is sparingly soluble in water, but may be given in a cachet or as a pill.

**Chronic Bronchitis.**

The following is a useful prescription:—Tinct. nux vomica, tinct. of sanguinaria, of each, min. xl; extract of pinus canadensis (dark), ʒiv; simple syrup, ʒiv. A teaspoonful every four hours.

**Chian Turpentine in Cancer.**

The use of chian turpentine in cancer of the uterus was suggested by Dr. Clay. Though other observers failed to get equally good results, most admitted that the drug certainly relieved the symptoms and especially the pain.

**Obstinate Hiccough.**

Dr. Charles S. Dickinson reports an obstinate case of hiccough which resisted a long series of remedies. At last it occurred to him to try the effect of oatmeal porridge served with milk, after which the hiccough promptly ceased. The suggestion is that the oatmeal had a demulcent action on the irritated gastric mucous membrane.

**Caffein.**

Caffein is not only a heart tonic, but is also a valuable diuretic. It may be prescribed as the citrate, and either alone or in combination with tincture of convallaria. One of its chief uses is in the slow, labouring heart of senility, as it somewhat accelerates the heart's action and invigorates the systole.—*Dr. Seymour Taylor.*

**Rickets and Caries of the Spine.**

The rigidity of the back at once separates cases of caries from the curvatures we so often find depending on rickets. In any doubtful case the child has only to be laid prone upon its chest, while the surgeon seizes its thighs and moves them laterally to and fro. In rickets the spine will be noticed to be quite mobile, but in caries it will move as a whole.—*Mr. Ballance.*

**Hæmorrhage after the Menopause.**

Always take it as a most serious symptom if a woman consults you, after the menopause, suffering from hæmorrhage. Always insist on a vaginal examination, and do not be deluded because you find the os and cervix normal, but thoroughly examine bi-manually, and if there is much pain in the examination do not hesitate to make a thorough examination under an anæsthetic.—*Mr. Bowreman Jessett.*

**Strychnine in Progressive Muscular Atrophy.**

In progressive muscular atrophy the hypodermic injection of strychnine is of unquestionable value. *Sir Wm. Gowers.*

**Duodenal Ulcer.**

If we find a patient who complains of pain rather to the right side of the abdomen, always coming on some hours after food, and if he passes black motions, we may be justified in assuming the probability of duodenal ulcer.—*Dr. Cayley.*

**Rectal Examination.**

It is wise never to prescribe for rectal symptoms until a thorough examination has been made. The neglect of this often means that the early, and therefore operable, stage of malignant disease is overlooked.

**Taxis in Strangulated Hernia.**

I would strongly enjoin on you that success in strangulated hernia is greatest for him who operates early and with a precise appreciation of the circumstances. I hold strongly to the view that a really strangulated hernia is rarely reducible by taxis.—*Mr. J. W. Hulke.*

**Thyroid Gland in Psoriasis.**

Dr. Byrom Bramwell believes that in this condition the best method is to give the patient as large doses as he can bear. He finds that many such patients can take fully 30 grains daily for long periods of time. The pulse-rate affords the best warning of excess in dose.

**Ulcer of the Œsophagus.**

Ulceration of the œsophagus is in the great majority of cases, to the extent probably of 90 per cent., due to carcinoma. It may, of course, result from the swallowing of corrosive liquids, and sometimes from the swallowing of a solid body. Syphilitic ulcers are described, but they are very rare.

**Spectacles for Children.**

The common apprehension that the wearing of spectacles by young people is fraught with danger, owing to a risk of breakage during play, and of incidental damage to the eyes by particles of glass, is very ill-founded and mischievous. I have met with no example of such an accident. Parents and teachers often demur to glasses being worn when cricketing, but certainly upon insufficient grounds. *Professor McHardy.*

**Capillary Bronchitis of Infants.**

This condition, unless arrested somehow, will soon end in death. The blue face and ear-tips, respiration reaching 100 or more, flying pulse with epigastric recession and struggling for breath, are significant enough. I know of no means of meeting this perilous state—and I have tried many—which can be compared with bleeding—bleeding by the application of leeches to the chest, not one or two, but four or five or six. This is to be followed by surface stimulation, or in the exceptional occurrence of hyperpyrexia, by the cautious application of ice. Of course, a large number of cases treated in this fashion die. But of all attempts at rescue I believe this to be the best.—*Dr. Octavius Sturges.*

